# Prevalence of depression and its correlative factors among female adolescents in China during the coronavirus disease 2019 outbreak

**DOI:** 10.1186/s12992-020-00601-3

**Published:** 2020-07-28

**Authors:** Jiaojiao Zhou, Xiaofei Yuan, Han Qi, Rui Liu, Yaqiong Li, Huanhuan Huang, Xu Chen, Gang Wang

**Affiliations:** The National Clinical Research Center for Mental Disorders & Beijing Key Laboratory of Mental Disorders, Beijing Anding Hospital & the Advanced Innovation Center for Human Brain Protection, Capital Medical University, No. 5 Ankang Hutong, Xicheng District, Beijing, 100035 China

**Keywords:** Prevalence, Depression, Female, Adolescent, COVID-19, Outbreak

## Abstract

**Background:**

The outbreak of 2019 coronavirus disease (COVID-19) could increase the risk of depression. However, epidemiological data on outbreak-associated depressive morbidity of female adolescents are not available. This study determines the incidence and correlates of depression among female adolescents aged 11–18 years during the COVID-19 outbreak in mainland China.

**Methods:**

A large cross-sectional sample, nationwide online survey was conducted during the COVID-19 outbreak. Depression was assessed using the Center for Epidemiologic Studies Depression Scale (CES-D), and the correlative factors of depression were analyzed.

**Results:**

In this study, 4805 female adolescents were enrolled with a median (range) age of 15 (11–18) years. Of them, 1899 (39.5%) suffered from depression with a CES-D score of > 15. The onset of depression was significantly related to age, grade, distant learning, attitude toward COVID-19, sleep duration, and physical exercise duration. Furthermore, participants aged 15–18 years (OR = 1.755, 95% CI: 1.550–1.987, *p* < 0.001), participating in distant learning (OR = 0.710, 95% CI: 0.564–0.894, *p* = 0.004), concerned about COVID-19 (OR = 0.414, 95% CI: 0.212–0.811, *p* = 0.010), with sleep duration/day of < 6 h (OR = 2.603, 95% CI: 1.946–3.483, *p* < 0.001),and with physical exercise duration/day < 30 min (OR = 1.641, 95% CI: 1.455–1.850, *p* < 0.001) represented to be independent factors for suffering from depression.

**Conclusion:**

During the COVID-19 outbreak, depression was common among female adolescents. Older age, distant learning, concern about COVID-19, short sleep duration, and physical exercise duration represented the independent factors for suffering from depression.

## Introduction

Depression is a common mental disorder among adolescents [1]. A study of 9586 Taiwanese adolescents in the community found that the prevalence of significant depression was 12.3% [2]. A meta-analysis of 51 studies involving 144,060 secondary school students in mainland China indicated an estimated incidence of depression of 24.3% [3]. One of the best replicated epidemiological findings in the study of depression is that females have higher prevalence of depressive symptoms than males during adolescence [4, 5]. The change in rates of depression therefore occurs at the same time as the hormonal and physical changes of puberty, and the many psychological, behavioral and social transitions that accompany them. This coincidence of timing has, not unnaturally, led to the suggestion that the physiological changes of puberty may be responsible in some way for the change in rates of depression. Depressive disorder was demonstrated as the leading cause of disability-adjusted life years in people aged 10–19 years [6]. Thus, early prevention, detection, and treatment are essential to protect female adolescents from depression and improve their mental health.

At the end of 2019, the corona virus disease 2019 (COVID-19), believed to have started from Wuhan, Hubei Province, China, has been of great concern nationwide and globally [7, 8]. It was declared a pandemic by the World Health Organization (WHO) on March 11, with the number of patients with COVID-19 and associated deaths increasing rapidly over the last several months [9].

The outbreak of COVID-19 had a significant negative impact on daily life and on the study of mental disorders, especially depression, among female adolescents [10], due to negative life event, heavy academic pressure, and low self-esteem, resulting in high risk of depressive symptoms, such as sense of fear, uncertainty, boredom, anger, and loneliness associated with quarantine and challenges due to conflict with parents, changes in learning methods, study pressure, and insufficient outdoor activities. The outbreak of COVID-19 has raised considerable challenges for mental health services among female adolescents. It is the responsibility of all stakeholders, from parents to governments, to ensure that the physical and mental impact of the COVID-19 outbreak on female adolescents is minimal [9]. Immediate action is needed.

To date, however, epidemiological data on outbreak-associated depressive morbidity among female adolescents is not available. Hence, it is necessary to conduct a large, cross-sectional nationwide online survey about outbreak-associated depression among female adolescents, and the purpose of this study was to determine the incidence and correlates of depressive symptoms among female adolescents aged 11–18 years during the COVID-19 outbreak in mainland China.

## Methods

### Study design and participants

This was a cross-sectional, national online survey conducted between February 20 and 27, 2020, based on the collaborative research network of the National Clinical Research Center for Mental Disorders, China. Using a snowball sampling during the COVID-19 outbreak in China, data were collected with the WeChat-based Wenjuanxing program (https://www.wjx.cn/). In most secondary schools in China, the WeChat program has been widely used for student management. The inclusion criteria were as follows: (1) female secondary school students aged between 11 and 18 years and; (2) living in mainland China during the COVID-19 epidemic. Participants who completed the questionnaire in less than 120 s or were not aged 11–18 years were excluded. All participants and their guardians provided written informed consent. This study was approved by the Medical Ethical Committee in Beijing Anding Hospital of the Capital Medical University, China.

### Assessment instruments and data collection

In this study, a data collection sheet was used to collect socio-demographic and clinical characteristics such as age, grade, participating in distant learning, study duration/day, parents as frontline workers, having infected acquaintances, attitude toward COVID-19, sleep duration/day, physical exercise duration, residence, and presence of depression. Based on the report of the National Health Commission of China (http://www.nhc.gov.cn), study sites were classified according to the total number of COVID-19 patients at the provincial level on February 27, 2020.

The presence of depression was assessed by the Center for Epidemiological Studies Depression Scale (CES-D), Chinese version [11], which was developed for use in surveys of depressive symptomatology in the general population and was a short, structured self-report scale [12]. The items of the scale are symptoms correlated to depression used in other validated longer scales [12]. The CES-D scale proved to be a reliable and measure valid for use with adolescents [13], and the symptoms could be grouped into four factors: depression, positive effect, somatic symptoms, and interpersonal problems (7,4, 7, and 2 items, respectively) [12]. Total scores range from 0 to 60, and a CES-D cut-off score of 16 had a sensitivity of 100% and a specificity of 76% [14].

### Statistical analyses

All statistical tests were performed with SPSS 22.0 software (IBM Corp, Armonk, NY, USA). Statistical significance was set as a *p*-value < 0.05 (two-tailed). Pearson’s test and chi-square test were used to determine the distribution of categorical variables, and Mann–Whitney *U* test was used for continuous variables. Univariate and multivariate analyses were performed to examine the correlates of depression using the logistic regression model.

## Results

### Sample characteristics and prevalence of depression

In this study, all provinces of mainland China were included, and 5052 female adolescents were invited to participate in the survey. Two hundred forty-seven participants were excluded from this study as they completed the questionnaire in less than 120 s (*n* = 67) or were not 11–18 years old (*n* = 180). Finally, 4805 female adolescents were enrolled with a median (range) age of 15 (11–18) years. Of them, 1899 (39.5%) suffered from depression with a CES-D score of > 15. Table 1 shows the socio-demographic and clinical characteristics between depression and no depression groups. We found that depression was more common among female adolescents who were 15–18 years old (30.8% vs. 46.3%, *p* < 0.001),in senior secondary school (33.5% vs. 47.1%, *p* < 0.001), not participating in distant learning (38.4% vs. 54.0%, *p* < 0.001), living in provinces where number of infected patients was < 1000 (35.8% vs. 40.2%, *p* = 0.020), not concerned about COVID-19 (39.3%vs. 65.0%,*p* = 0.001), had sleep duration/day of < 6 h (64.7%vs. 41.4% vs. 33.2%, *p* < 0.001), or had physical exercise duration/day of < 30 min (46.4% vs. 32.7% vs. 34.4%, *p* < 0.001). There was no notable difference in the prevalence among the subgroups divided by study duration/day, parents as frontline workers, having infected acquaintances, and residence.

**Table 1:** Demographic characteristics of the study sample (*n* = 4805)

Variables	Total, n (%)	Depression		*p* value
		No, n (%)	Yes, n (%)	
Age, year				< 0.001*
11–14	2114 (44.0)	1462 (69.2)	652 (30.8)	
15–18	2691 (56.0)	1444 (53.7)	1247 (46.3)	
Grade				< 0.001*
Junior secondary school	2692 (56.0)	1789 (66.5)	903 (33.5)	
Senior secondary school	2113 (44.0)	1117 (52.9)	996 (47.1)	
Participating in distant learning				< 0.001*
Yes	4459 (92.8)	2747 (61.6)	1712 (38.4)	
No	346 (7.2)	159 (46.0)	187 (54.0)	
Study duration/day, h				0.067
< 4	3254 (67.7)	1939 (59.6)	1318 (40.4)	
≥ 4	1551 (32.3)	967 (62.3)	584 (37.7)	
Parents as frontline workers				0.345
Yes	415 (8.6)	242 (58.3)	173 (41.7)	
No	4390 (91.4)	2664 (60.7)	1726 (39.3)	
Having infected acquaintances				0.536
Yes	160 (3.3)	93 (58.1)	67 (41.9)	
No	4645 (96.7)	2813 (60.6)	1832 (39.4)	
No. of infected patients at living province				0.020*
< 1000	4031 (83.9)	2409 (59.8)	1622 (40.2)	
≥ 1000	774 (16.1)	497 (64.2)	277 (35.8)	
Concerned about COVID-19				0.001*
Yes	4765 (99.2)	2892 (60.7)	1873 (39.3)	
No	40 (0.8)	14 (35.0)	26 (65.0)	
Sleep duration/day, h				< 0.001*
< 6	218 (4.5)	77 (35.3)	141 (64.7)	
6–8	2854 (59.4)	1672 (58.6)	1182 (41.4)	
> 8	1733 (36.1)	1157 (66.8)	576 (33.2)	
Physical exercise duration/day (indoor and outdoor), min				< 0.001*
< 30	2364 (49.2)	1268 (53.6)	1096 (46.4)	
30–60	2127 (44.3)	1432 (67.3)	695 (32.7)	
> 60	314 (6.5)	206 (65.6)	108 (34.4)	
Residence				0.07
Dorm	4215 (87.7)	2529 (60.0)	1686 (40.0)	
No dorm	590 (12.3)	377 (63.9)	213 (36.1)	

* Statistically significant

### Characteristics associated with depression

Univariate logistic regression analysis in this study showed that participants aged 15–18 years (odds ratio [OR] =1.936, 95% CI: 1.718–2.182, *p* < 0.001), with sleep duration/day of < 6 h (OR = 2.947, 95% CI: 2.218–3.915, *p* < 0.001), and with physical exercise duration/day of < 30 min (OR = 1.763, 95% CI: 1.569–1.982, *p* < 0.001) had higher odds of suffering from depression (Table 2). Those who participated in distant learning (OR = 0.530, 95% CI: 0.425–0.660, *p* < 0.001), lived in provinces with number of infected patients ≥1000 (OR = 0.828, 95% CI: 0.705–0.971, *p* = 0.020), and were concerned about COVID-19(OR = 0.349, 95% CI: 0.182–0.670, *p* = 0.002) were less likely to develop depression (Table 2). In the multivariate logistic regression model, participants aged 15–18 years (OR = 1.755, 95% CI:1.550–1.987, *p* < 0.001), participating in distant learning (OR = 0.710, 95% CI:0.564–0.894, *p* = 0.004), concerned about COVID-19 (OR = 0.414, 95% CI:0.212–0.811, *p* = 0.010), with sleep duration/day of < 6 h (OR = 2.603, 95% CI:1.946–3.483, *p* < 0.001), and physical exercise duration/day of < 30 min (OR = 1.641, 95% CI:1.455–1.850, *p* < 0.001) represented the independent factors for suffering from depression (Table 2).

**Table 2:** Univariate and multivariate logistical regression analyses of correlates of depression

Variables	Univariate analyses		Multivariate analyses	
	OR(95%CI)	*p* value	OR(95%CI)	*p* value
Age, 15–18 vs 11–14	1.936 (1.718–2.182)	< 0.001*	1.755 (1.550–1.987)	< 0.001*
Participating in distant learning, yes vs no	0.530 (0.425–0.660)	< 0.001*	0.710 (0.564–0.894)	0.004*
Study duration/day, ≥4 h vs < 4 h	0.891 (0.786–1.008)	0.067	0.949 (0.833–1.080)	0.425
Parents as frontline workers, yes vs no	1.103 (0.899–1.354)	0.345	1.108 (0.897–1.370)	0.341
Having infected acquaintances, yes vs no	1.106 (0.804–1.523)	0.536	1.028 (0.740–1.429)	0.869
No. of infected patients at living province, ≥1000 vs < 1000	0.828 (0.705–0.971)	0.020*	0.881 (0.747–1.038)	0.131
Concerned about COVID-19, yes vs no	0.349 (0.182–0.670)	0.002*	0.414 (0.212–0.811)	0.010*
Sleep duration/day, < 6 h vs ≥6 h	2.947 (2.218–3.915)	< 0.001*	2.603 (1.946–3.483)	< 0.001*
Physical exercise duration/day, < 30 min vs ≥30 min	1.763 (1.569–1.982)	< 0.001*	1.641 (1.455–1.850)	< 0.001*
Dorm residence, yes vs no	1.180 (0.987–1.411)	0.070	1.169 (0.972–1.405)	0.098

* Statistically significant

### Four factors of CES-D scale of female adolescents with depression

The mean total CES-D score was 26.43 ± 8.54 among the 1899 female adolescents with depression. Female adolescents with depression in the age group of 15–18 years had higher scores in positive effect (4.77 ± 2.69 vs. 4.35 ± 2.66, *p* = 0.001), somatic symptoms (8.38 ± 3.20 vs. 7.74 ± 3.12, *p* < 0.001), and total score (26.90 ± 8.54 vs. 25.52 ± 8.47) than those in the age group of 11–14 years. However, there was no notable difference in the scores of depression and interpersonal problems between the subgroups divided by age (Table 3).

**Table 3:** Four factors of CES-D Scale of female adolescents with depression in the subgroups divided by age

Variables	Age groups		*p* value
	11–14	15–18	
Depressed affect	11.07 ± 4.19	11.33 ± 4.33	0.207
Positive affect	4.35 ± 2.66	4.77 ± 2.69	0.001*
Somatic symptoms	7.74 ± 3.12	8.38 ± 3.20	< 0.001*
Interpersonal problems	2.36 ± 1.65	2.42 ± 1.66	0.486
Total score	25.52 ± 8.47	26.90 ± 8.54	0.001*

* Statistically significant

## Discussion

Female adolescents are vulnerable to depression [10]. The outbreak of COVID-19 had a significant negative impact on daily life and on the study of female adolescents, resulting in increased risk of depression. Chinese is the largest ethnic group comprising one-fifth of the world population and has a larger number of female adolescents than other countries. Early prevention, detection, and treatment are essential to protect female adolescents from depression and improve their mental health. In this national online cross-sectional survey, we found that 39.5% of the female adolescents suffered from depression during the COVID-19 outbreak, and the onset of depressive symptoms was significantly related to age, grade, distant learning, attitude toward COVID-19, sleep duration/day, and physical exercise duration. Furthermore, our results indicated that older age, participating in distant learning, concerned about COVID-19, sleep duration/day of < 6 h, and physical exercise duration/day of < 30 min represented the independent factors for suffering from depression. This fact maybe highly informative for all stakeholders, from parents to governments, to develop intervention efforts targeting high-risk groups.

A recent systematic review and meta-analysis of 144,060 Chinese secondary school students indicated that the pooled prevalence of depression was 24.3% (95% CI: 21.3–27.6) [3]. In this study, we found a higher depression prevalence, which means that the COVID-19 outbreak is a vital risk factor of depression for female adolescents. The emergence of COVID-19 has parallels with the outbreak of severe acute respiratory syndrome, which killed 349 of 5327 infected patients in China [15]. As an unpleasant experience, the COVID-19 outbreak led to mass quarantine; fears of infection; boredom; anger; frustration; lack of contact with friends, classmates, and teachers; and lack of family, finance, and personal space at home, all of which are associated with increased risk of depression [9, 16, 17]. Since the COVID-19 epidemic was no longer confined to China, problems related to school closure and home confinement also became relevant in other affected countries.

The Government of China ordered a nationwide school closure to prevent the spread of COVID-19 and public activities were discouraged. Millions of adolescents were confined to their homes, resulting in decreased visits to others and outdoor activities. Prolonged school closure and home confinement during the COVID-19 outbreak might have a negative impact on children’s mental health, although these efforts and measures were highly necessary and commendable. We also found that less physical exercise and sleep duration were significantly associated with higher risk of depression, which was consistent with the findings of previous studies. Physical exercise proved to be a promising antidepressant treatment for adolescents aged 13–17 years [18]. We found that light to moderate intensive exercise three times a week for 6–12 weeks could bring alleviate depression [18]. Existing studies suggested that the relationship between short sleep duration and depression was bidirectional [19]. Accumulating evidence suggested that short sleep duration might be a prospective predictor of depressive symptoms among adolescents [20].

Our results indicated that female adolescents aged 15–18 years had higher risk of depression and higher CES-D scores. Female adolescents aged 15–18 years old had higher risk of severe depressive symptoms, considering higher scores indicating a greater number and increased frequency of depressive symptoms. In this study, we found that senior secondary school students had higher risk of depression than junior secondary students. After graduating from junior secondary school, most students in China entered secondary schools. These adolescents faced enormous academic pressure due to college entrance examinations, which was described as a stampede of “thousands of soldiers and tens of thousands of horses across a single log bridge” [3]. This may have led to the findings.

In this survey, female adolescents concerned more about the COVID-19 outbreak had lower risk of depression. This is partly because clear communication and regular and accurate updates about COVID-19 could improve relevant knowledge of the pandemic and reduce the sense of uncertainty and fear [7]. Additionally, the National Health Commission of China released the national guideline of psychological crisis intervention for COVID-19, which could help female adolescents better understand COVID-19. In brief, concerns about the COVID-19 outbreak were related to lower risk of depression for decreased sense of uncertainty and fear. Interestingly, the participants from the provinces of lower number of reported COVID-19 cases were more likely to score high on depressive symptoms. Partly because female adolescents concerned less about the COVID-19 outbreak, resulting in increased sense of uncertainty and fear. Future studies focus on those so-called counterintuitive results are needed.

Several limitations should be noted in this study. First, this is an online study; therefore, those with no access to the Internet could not join. However, distance teaching has been used nationwide during the COVID-19 outbreak, and only very few adolescents had no access to the Internet. Second, some data pertaining to important factors associated with depression, such as physical health and social support, were not available due to logistical reasons. Finally, due to the cross-sectional study design, causality between variables could not be examined. Hence, a well-designed study of the prevalence of depression is necessary to complement epidemiological studies and provide more conclusive evidence regarding the incidence of depression and its correlative factors among female adolescents during the COVID-19 outbreak.

## Conclusion

Depression was common among female adolescents during the COVID-19 outbreak. Older age, distant learning, concern about COVID-19, short sleep duration, and physical exercise duration represented the independent factors for suffering from depression. Considering the negative impact of depression, timely screening and appropriate interventions are urgently needed for depressed female adolescents during the COVID-19 outbreak.

## Data Availability

The data used in this this study are available from the corresponding author upon reasonable request.
